# Bronchoalveolar lavage fluid in immune checkpoint inhibitor-related pneumonitis: from pathophysiological window to a precision diagnostic tool

**DOI:** 10.3389/fimmu.2026.1788186

**Published:** 2026-02-18

**Authors:** Zheng Linpeng, Yang Jing, Qi Yaxian, Lin Fenglin, Chen Xiewan, Sun Jianguo

**Affiliations:** 1 Cancer Institute, Xinqiao Hospital, Army Medical University, Chongqing, China; 2 Department of Thoracic Oncology Center, Chongqing University Cancer Hospital, Chongqing, China; 3 Department of Basic Medicine, Army Medical University, Chongqing, China

**Keywords:** BALF (bronchoalveolar lavage fluid), biomarkers, CIP, ICIs - immune checkpoint inhibitors, single-cell sequence (ScRNA-seq)

## Abstract

Immune checkpoint inhibitor related pneumonitis is a serious adverse reaction with diverse clinical and radiologic patterns, which make both diagnosis and treatment challenging. Therefore, understanding its underlying biology is crucial for improving clinical management. Bronchoalveolar lavage fluid provides a minimally invasive way to explore the lung immune environment, and it supports cytologic, molecular, and multi-omics analyses. In particular, BALF lymphocytosis serves as a key diagnostic sign. Furthermore, single-cell sequencing has revealed that abnormal T-cell activation and myeloid cell reprogramming play central roles in the development of CIP. These findings have, in turn, led to the identification of potential biomarkers such as CCL18, IL-6, and IP-10 for early detection and disease monitoring. However, the absence of standardized sampling and interpretation methods still limits the reproducibility of results and the broader application of BALF analysis in clinical practice. In the future, integrating BALF-derived data into artificial intelligence frameworks may enhance diagnostic precision and guide personalized therapy. Overall, BALF represents a valuable platform for refining CIP classification, clarifying disease mechanisms, and supporting the development of targeted treatments.

## 1 Introduction

**Figure 1:**
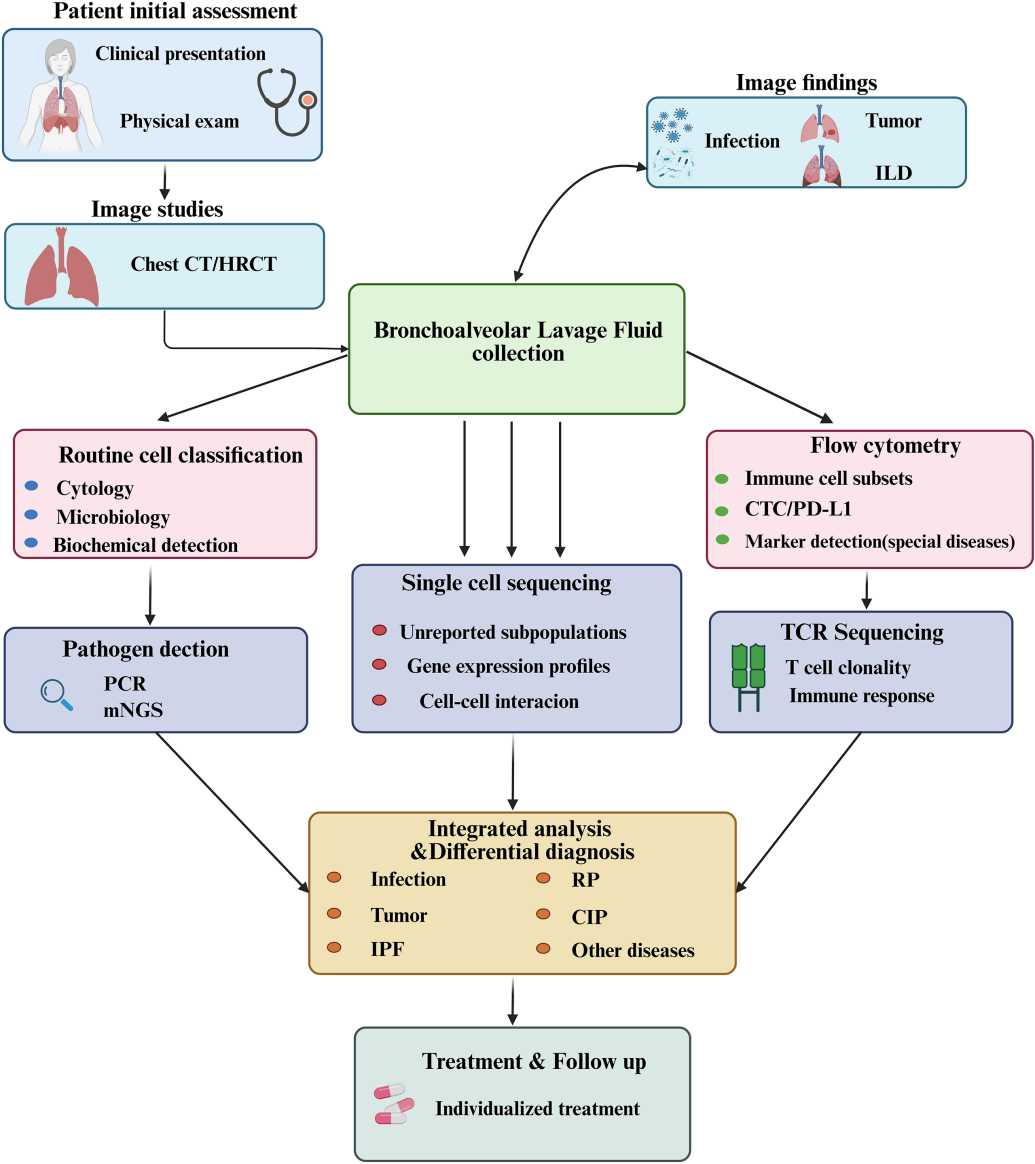
The application of BALF in the diagnosis and prognosis of lung diseases. ILD, Interstitial Lung Diseases; HRCT, High-resolution Computed Tomography; CTC, Circulating Tumor Cell; PCR, Polymerase Chain Reaction; mNGS, metagenomic Next-generation Sequencing; RP, Radiation Pneumonitis; CIP, Checkpoint Inhibitor-Related Pneumonitis; IPF, Idiopathic Pulmonary Fibrosis.

Immune checkpoint inhibitors (ICIs) have become a cornerstone of modern oncology, significantly improving survival in patients with a wide range of cancers (1). However, their use is frequently accompanied by immune-related adverse events, among which checkpoint inhibitor-related pneumonitis (CIP) has emerged as a major concern due to its potentially life-threatening nature (2–4). Although CIP occurs in a relatively small proportion of patients, it remains the leading cause of immunotherapy-related mortality (3). Furthermore, real-world data suggest that its incidence is higher than what has been documented in clinical trials (2). Taken together, these observations underscore an urgent need for accurate and timely diagnosis of CIP in routine practice. In this regard, the diagnostic process is complicated by the considerable heterogeneity of CIP manifestations, which can range from asymptomatic radiologic findings to severe respiratory failure (3, 5). At the same time, its variable and often nonspecific imaging patterns make clinical interpretation challenging (2, 6). In patients with cancer, new pulmonary infiltrates may have multiple causes, including infections, tumor progression, radiation pneumonitis, drug-related lung injury from targeted therapies, or pre-existing interstitial lung disease (2, 7, 8). Consequently, the imaging features of CIP substantially overlap with those of these conditions (2, 6), and differentiation based solely on clinical and radiologic criteria is frequently unreliable (7, 9). Moreover, lung tissue sampling, although offering definitive histopathologic confirmation, is often not feasible in patients who are critically ill or at high risk for complications. To address these challenges, bronchoalveolar lavage fluid (BALF) analysis has attracted increasing attention as a minimally invasive approach for characterizing immune and inflammatory activity within the pulmonary microenvironment. In particular, BALF enables direct, *in situ*, and dynamic assessment of local immune responses, providing obvious advantages over serum biomarkers and imaging-based modalities (10). Historically, BALF has been used in the evaluation of interstitial lung diseases, and it continues to provide valuable insights into underlying pathophysiology by identifying cellular subsets, soluble mediators, metabolites, and microbial communities (11–15). Recently, researchers have applied advanced techniques such as single-cell RNA sequencing, T-cell receptor sequencing, metabolomic profiling, and microbiome analysis to BALF samples from patients with CIP. Through these approaches, several distinct immunologic patterns have been identified. In particular, BALF samples from patients with CIP show increased proportions of both CD4^+^ and CD8^+^ T cells. Additionally, there is a notable expansion of follicular helper-like CD4^+^ T cells, characterized by strong inflammatory activity and clear clonal proliferation features. At the same time, an accumulation of regulatory T cells with enhanced suppressive function has been observed, while effector memory and tissue-resident memory CD8^+^ T cells appear particularly enriched (16). Moreover, complementary studies have examined BALF characteristics such as lymphocytosis, cytokine profiles, metabolite signatures, and microbial composition as potential biomarkers for CIP (11, 13, 15). As a result, BALF-based investigations have greatly advanced current understanding of CIP immunopathogenesis. Overall, these findings indicate that BALF analysis holds substantial promise not only for elucidating disease mechanisms but also for overcoming major diagnostic limitations associated with this complex and potentially fatal condition (**Figure 1**). Although direct evidence from BALF studies specifically focused on CIP remains limited, and reported BALF features vary across studies (17), extensive experience and well-established methodologies from its application in other pulmonary disorders, such as chronic hypersensitivity pneumonitis, idiopathic pulmonary fibrosis, and sarcoidosis (14, 18, 19), provide a robust theoretical and technical foundation for exploring its role in a disease as complex and challenging as CIP.

## 2 Clinical applications of BALF in common pulmonary disorders

### 2.1 BALF in infectious diseases

BALF is vital for diagnosing bacterial, fungal, viral, and pneumocystis pneumonia (PCP) (20–24). Molecular analyses enable sensitive pathogen detection; metagenomic sequencing identifies diverse organisms, aiding rare or mixed infection diagnosis. BALF also distinguishes microbiota profiles in PCP and reveals relevant biomarkers (25). Galactomannan detection supports invasive aspergillosis diagnosis with higher sensitivity than serum tests (26), while cytological and molecular tests improve cytomegalovirus (CMV) detection (27). In immunocompromised or AIDS patients, BALF identifies multiple pathogens such as Mycobacterium tuberculosis (28, 29), and PCR aids recognition of Legionella spp (30). Overall, as a minimally invasive tool, BALF integrates culture and molecular assays to enhance early and differential diagnosis of pulmonary infections (31).

### 2.2 BALF analysis for inflammatory cell types

Analyzing inflammatory cells in BALF is important for understanding lung inflammation and helping to identify different types of hypersensitivity pneumonitis and interstitial lung diseases. Apart from CEP (Chronic Eosinophilic Pneumonia) and AEP (Acute Eosinophilic Pneumonia), eosinophilia in BALF can also be suggestive of allergic disease or asthma (32), linked to Th2 cytokines IL−4 and IL−5 (33, 34). Lymphocytosis indicates chronic hypersensitivity pneumonitis or connective tissue disease−associated ILD (35, 36), with CD4^+^ T−cell predominance in sarcoidosis and neutrophilia in IPF (35, 37). Neutrophilia also appears in bacterial infection and acute lung injury (38, 39). Integrated with clinical data, BALF cytology helps predict progression, guide therapy (36, 37), and reduce the need for tissue biopsy (40, 41).

### 2.3 Cytology and flow cytometric analysis of BALF

Cytological and flow cytometric analyses of BALF are useful for diagnosing both malignant and immune−mediated diseases by identifying malignant cells and characterizing immune cell subsets, including pulmonary involvement in hematologic malignancies. Conventional cytology using HE−stained smears or cell blocks can detect tumor cells but has limited sensitivity for pulmonary metastases. Flow cytometry allows rapid phenotyping of T, B, and myeloid cells (42–44), supports ILD assessment by analyzing the CD4^+^/CD8^+^ ratio (40, 44), and provides high−throughput, reproducible results (42). When combined with immunofluorescence or electron microscopy, FCM can also uncover inflammatory mechanisms such as neutrophil extracellular traps (45). Although not intended as a primary cancer screening tool, the combination of BALF cytology and FCM improves detection of metastases and evaluation of ICI responses, thereby enhancing diagnostic accuracy in pulmonary diseases (40, 44).

### 2.4 Molecular diagnostic applications of BALF

PCR and mNGS testing of BALF allow for fast and accurate identification of pathogens. PCR can detect specific organisms such as legionella and pneumocystis, helping to reduce misdiagnosis (46). mNGS provides unbiased detection of mixed or rare infections, including pneumocystis, influenza virus, and aspergillus, which is especially useful in immunocompromised patients (47). When combined with BALF cytology, it can also assist in diagnosing pediatric PCP with CMV (27). In addition, mNGS can speed up antimicrobial susceptibility testing to about 200 minutes (46). In clinical practice, these tools are valuable for differentiating infectious from non-infectious conditions and for clarifying the cause of pneumonia in immunocompetent individuals (47).

### 2.5 Immune mediators testing in BALF

BALF immune mediators—cytokines, chemokines, and immunoglobulins—serve as biomarkers of pulmonary inflammation and severity. Cytokines such as TNF−α, IL−6, IL−4, and IL−13 indicate inflammatory status; IL−4/IL−13 elevations relate to eosinophilic asthma, while reduction reflects therapy response (48, 49). Chemokine ratios, such as CCL7/CCL22, can predict mortality in ARDS (50), and shifts in mediator patterns during Candida albicans infection suggest changes in immune regulation (51). IgE levels are associated with allergic airway hyperreactivity (33, 34), whereas increases in TNF−α and IL−6 signal oxidative stress and disease progression (39). Interventions like curcumin nanoemulsion have been shown to reduce IL−4, IL−6, and IL−13, highlighting resolution of inflammation (48). Overall, immune profiling of BALF provides important insights for understanding, tracking, and managing pulmonary diseases.

In summary, BALF serves as a versatile and informative tool in the comprehensive management of pulmonary diseases. By employing molecular techniques such as PCR and mNGS, clinicians can substantially enhance pathogen detection and gain deeper insights into the underlying infectious processes. At the same time, cellular and cytometric analyses provide valuable information that aids in distinguishing interstitial, allergic, and neoplastic disorders. Furthermore, the profiling of immune factors enables a precise quantitative assessment of inflammatory activity, thereby supporting the development of individualized therapeutic strategies. Taken together, as a minimally invasive diagnostic approach, BALF unites multiple analytical modalities to improve diagnostic accuracy and promote more informed and effective clinical decision-making.

## 3 Features and diagnostic challenges of CIP

CIP is one of the most common immune−related adverse events (irAEs) that occur during treatment with ICIs, characterized by a relatively high incidence, diverse clinical manifestations, and considerable severity (52, 53). Notably, the incidence of CIP in real−world settings appears higher than that reported in clinical trials, with approximately 0–10% of patients developing pneumonitis of any grade (54). Among individuals with non−small cell lung cancer (NSCLC), the incidence is around 5%, and 1–2% may experience severe (grade ≥ 3) disease (55, 56). Although relatively uncommon, CIP can be life threatening and represents one of the leading causes of ICI−related mortality, with a reported death rate of up to 20% (56). Clinically, presentations vary widely from asymptomatic cases to severe manifestations, including dyspnea, cough, and hypoxemia. Importantly, some cases may mimic tumor metastasis or acute exacerbation of chronic obstructive pulmonary disease (COPD), thereby complicating recognition (57–60). CIP can emerge both early and late in the course of treatment and may even develop after discontinuation of ICIs. Moreover, a proportion of cases occur in patients with advanced malignancies, highlighting the need for sustained clinical vigilance (60). Risk factors include T−cell subset dysregulation, with a higher prevalence observed among NSCLC patients (55, 61). The diagnostic challenges of CIP are multifaceted. First, diagnosis primarily relies on clinical presentation and imaging features; however, the absence of specific biomarkers often leads to misdiagnosis or delayed recognition (62, 63). In this context, imaging modalities such as chest computed tomography (CT) play a central role in detection and monitoring, revealing heterogeneous patterns that may include ground−glass opacities or consolidation (64). Nevertheless, these patterns frequently overlap with those of radiation pneumonitis or infectious pneumonia, and careful integration of clinical history is required to exclude alternative etiologies (65–67). When multiple overlapping factors coexist, the complexity of differential diagnosis increases substantially. Second, CIP symptoms are inherently nonspecific. For instance, dyspnea is a common symptom shared by various pulmonary conditions, such as thoracic malignancies and COPD, which further complicates differential diagnosis (68, 69). Moreover, late−onset cases, particularly those arising several months after treatment discontinuation, are at increased risk of being overlooked. Such delayed recognition may result in progressive deterioration of respiratory function and, in severe cases, even mortality (59, 60). Finally, the underlying pathophysiological mechanisms of CIP remain incompletely elucidated, and its histopathological characteristics are poorly defined, both of which limit diagnostic precision (70). Therefore, in routine clinical practice, heightened vigilance, timely imaging evaluation, and close multidisciplinary collaboration are critical for optimizing the management of this complex condition.

## 4 Applications of BALF in the diagnosis and mechanistic studies of CIP

### 4.1 BALF’s role in the differential diagnosis of CIP

The clinical and radiologic features of CIP often overlap with those of pulmonary infection, tumor progression, or other interstitial lung diseases (ILDs). Findings such as ground-glass opacities and areas of consolidation are not unique to CIP, which frequently leads to diagnostic uncertainty in practice (7). In such situations, analysis of BALF can be extremely useful as a complement to imaging. When chest CT reveals infiltrates that are difficult to classify, BALF culture and cytological examination can provide valuable clues and help clinicians distinguish drug-induced pneumonitis from other ILDs (71). Occasionally, CIP presents with rather nonspecific findings on imaging, for instance focal consolidation that could also suggest infection. In these cases, a BALF profile dominated by lymphocytes tends to support an immune-mediated process and may prevent misdiagnosis (72). More broadly, BALF analysis reflects the local pulmonary immune environment and often provides direct evidence supporting a diagnosis of CIP. Lymphocytosis is particularly notable; several studies have documented lymphocyte fractions exceeding 20% in BALF from affected patients, whereas neutrophilia is more commonly associated with bacterial pneumonia (13). Interestingly, one study reported that a lymphocyte fraction greater than 25% had an 82% sensitivity and 76% specificity for CIP (13). Retrospective observations further suggest that increased lymphocytes correlate with disease severity, and that a CD4/CD8 ratio above one may predict recurrence (73). Moreover, BALF-based mNGS together with cultures for bacteria, fungi, and viruses plus cytological evaluation for malignant cells remain vital for ruling out infection or tumor involvement (74). In addition to aiding differential diagnosis, BALF composition may offer an early impression of disease severity. This point is clinically important because CIP can behave quite unpredictably; mild cases sometimes deteriorate rapidly. Preliminary work from our group using ScRNA-seq indicated that BALF samples from severe CIP contained an expanded epithelial cell population responsible for intensive cytokine release and accumulation of neutrophils (75). Detection of this population by flow cytometry could serve as an early warning signal for impending severe pneumonitis and help guide treatment decisions. Taken together, serial assessment of BALF during the course of illness may provide dynamic, clinically relevant information and support individualized management strategies for CIP.

### 4.2 BALF as a window into CIP mechanisms

Traditional BALF analysis offers averaged results derived from all cells in the sample, which tends to obscure variations among distinct cell subpopulations. This limitation has been largely overcome with the development of ScRNA-seq. The technology allows researchers to explore gene expression profiles, epigenetic states, and immune receptor diversity at the single cell level (76). When applied to BALF specimens, ScRNA-seq enables a more refined understanding of immune cell heterogeneity within the lung, facilitates the identification of rare but potentially pathogenic cell types, and helps reconstruct communication networks among different immune and structural cells. Integration of such high-resolution sequencing with clinically collected BALF has greatly narrowed the gap between mechanistic research and bedside application, moving respiratory medicine toward more precise diagnostics and personalized care (77). Combining ScRNA-seq with immune phenotyping has revealed that changes in BALF immune cell populations over time may provide important clues to the pathogenesis of CIP.

#### 4.2.1 Dysregulated T cell activation and clonal expansion

BALF from patients with CIP frequently shows accumulation of follicular helper-like T cells. Transcriptomic studies demonstrate strong expression of inflammatory genes such as IFN−γ and TNF−α, which may facilitate B cells activation and promote antibody production. Meanwhile, the proportion of regulatory T cells appears increased, and their suppressive capacity may become more pronounced, possibly acting as a compensatory counterbalance to excessive inflammation (16). CD8^+^ T cells in BALF often display tissue-resident characteristics, with effector memory and resident subsets expanding and releasing perforin and granzyme continuously, directly damaging alveolar epithelium (16, 78). In line with these findings, another report described a distinct accumulation of CXCL13^+^ T cells accompanied by overactivation of CXCL9^+^ monocytes, jointly driving exaggerated inflammation in CIP (79). Serum concentrations of IL−17A and IL−35 have been reported to rise significantly at CIP onset compared with baseline and to decline after clinical improvement. This pattern suggests that levels of IL−17A and IL−35 in BALF might serve as indicators of disease activity and severity in patients with NSCLC undergoing immune checkpoint therapy (80). Apart from cytokine profiling, T cell receptor (TCR) sequencing has demonstrated oligoclonal T cell expansion within BALF, supporting the concept that antigen driven responses play a key role in the development of CIP (78, 79). However, the specific antigens responsible for triggering these reactions remain to be identified, and ongoing research aims to clarify this critical question.

#### 4.2.2 Myeloid cell changes

Recent studies have demonstrated that myeloid cells within the BALF of CIP patients show distinct phenotypic alterations. Specifically, proinflammatory monocyte−derived macrophages are markedly increased and exhibit strong expression of cytokines such as IL−1β and IL−6, both of which are closely associated with pulmonary tissue injury (80). In parallel, analysis of monocyte developmental trajectories in BALF has revealed that LAMP3^+^ dendritic cells (DCs), which originate from CXCL9^+^ monocytes, have the potential to migrate from tumor tissue into BALF. However, differentiation toward anti−inflammatory macrophages appears to be impaired in CIP. Importantly, several signaling pathways, including CXCL9/10/11–CXCR3, FASLG–FAS, and IFNGR1/2–IFNG, are highly activated in BALF samples from affected patients (79). Moreover, some reports have described an increased abundance of plasma cells in BALF, which may contribute to the production of autoantibodies and further aggravate parenchymal inflammation (81).

It should be noted that CIP is a dynamic process, with disease activity and immunological characteristics potentially changing over time. Consequently, BALF specimens obtained from patients at different stages of the disease may show significant variability, limiting direct comparison. Performing longitudinal BALF analyses can provide critical information on the underlying mechanisms of CIP and aid in predicting clinical outcomes.

### 4.3 Multi-omics BALF analysis in CIP diagnosis and management

In addition to the immune−cell alterations revealed by single−cell RNA sequencing in CIP, multi−omics analysis of BALF offers new opportunities to identify disease−specific biomarkers. Such approaches can, in turn, enhance diagnostic accuracy and assist in guiding clinical management (**Table 1**).

**Table 1:** Methods applied in BALF analysis for CIP.

Research method	Key finding	Ref No.	Strengths
Single-cell RNA sequencing	Immune Activation and Phenotypic Remodeling of T Cell and Myeloid Cell Subsets	(16, 75, 78, 79)	Revealing the fine heterogeneity of the immune microenvironment to identify potential therapeutic targets
T-cell receptor sequencing	Oligoclonal expansion of T cells	(78, 79)	Changes in the TCR repertoire can serve as prognostic biomarkers
Cytokine profiling	elevated levels of IL−6, IL−1β, IFN−γ and IP−10	(15, 82)	Revealing the immune status of CIP to support the development of targeted therapies
Metabolomics	Suppressed amino sugar metabolism, decreased spermidine and spermine biosynthesis, and enhanced metabolism of α−linolenic acid, linoleic acid, and related fatty acids were observed.	(11)	Identifying specific metabolic biomarkers and understanding the pathological processes of CIP.
mNGS	Reduced α diversity, decreased Streptococcus, and increased Proteobacteria and Firmicutes. Help differentiate CIP from severe pulmonary infection.	(12, 83, 84)	Rapid and accurate detection provides clinicians with timely etiological evidence
Proteomics	Elevated macrophage CCL18 expression	(85)	Capable of identifying differentially expressed proteins linked to the severity of CIP.
Existing predictive models	Featured by CXCL9, CXCL10, CXCL11, CXCL13, CXCR3, CXCR6, FASLG, and IFNG. Or BALF microbiota signatures	(12, 79)	A future direction of precision medicine that can assist clinicians in decision-making

#### 4.3.1 Cytokine profiling

Recent studies have shown that analyzing cytokine profiles together with immune−cell subset composition in BALF provides valuable information for the differential diagnosis of CIP. Specifically, inflammatory cytokines such as IL−6, IL−1β, and IFN−γ are significantly elevated in the BALF of CIP patients compared with those observed in the pulmonary infection group, whereas the anti−inflammatory cytokine IL−10 shows higher levels in Treg−enriched samples (82). Furthermore, another study demonstrated that IP−10 concentrations are markedly increased in the BALF of CIP patients, accompanied by an enrichment of CD8^+^ T cells (15).

#### 4.3.2 Metabolomics

Untargeted metabolomic analysis revealed that, compared with newly diagnosed lung cancer patients, CIP patients exhibit suppressed amino sugar metabolism and reduced biosynthesis of spermidine and spermine in BALF. In contrast, when compared with the idiopathic pulmonary fibrosis (IPF) group, the CIP group shows increased metabolism of α−linolenic acid, linoleic acid, and their fatty acid derivatives. This study also identified 12 BALF metabolites in CIP patients that positively correlated with the proportion of CD8^+^ T cells (11). These metabolites may potentially serve as predictive biomarkers for CIP onset; however, validation in larger cohorts is required.

#### 4.3.3 mNGS

Prospective studies have shown that the BALF microbiome composition in CIP patients differs markedly from that in individuals with pulmonary infections, with reduced α-diversity and altered abundance of specific genera such as *Streptococcus*, potentially linked to disease pathogenesis (12). Another retrospective study found that higher relative abundances of *Proteobacteria* phylum and *Firmicutes* phylum in lower respiratory BALF samples are significantly associated with CIP in lung cancer patients (83). Moreover, monitoring BALF via mNGS can assist in differential diagnosis. In a reported case, intensive care physicians employed mNGS to differentiate CIP from severe pulmonary infection, leading to successful life-saving management (84).

#### 4.3.4 Proteomics

One study reported that chemokine ligand 18 (CCL18) levels in macrophages from CIP patients were markedly elevated. This increase was consistently observed across multiple analytic layers, including the transcriptomic level assessed by single cell RNA sequencing, the cellular level evaluated by flow cytometry, and the secreted protein level measured by BALF ELISA. These findings showed a clear positive association with disease severity (85). Interestingly, similar proteomic alterations have been described in idiopathic pulmonary fibrosis (IPF), where significantly increased levels of S100A9 were detected in BALF (18).

#### 4.3.5 Predictive models

Cui and colleagues developed a predictive model for checkpoint inhibitor-related pneumonitis (CIP) that incorporated eight molecular features, namely CXCL9, CXCL10, CXCL11, CXCL13, CXCR3, CXCR6, FASLG, and IFNG, achieving an area under the receiver operating characteristic curve (AUC) of 0.755 (79). Building on a different analytical approach, Zhou and collaborators applied machine learning algorithms to BALF microbiota profiles in order to distinguish CIP from non-CIP cases. Notably, the decision tree method outperformed other models, reaching an AUC of 0.88 (12). Although these findings are encouraging, the number of available predictive models remains small, and their diagnostic accuracy is still suboptimal. This suggests a clear need for future work to design more robust and reliable tools that can enhance both diagnostic precision and prognostic evaluation in clinical practice.

Taken together, comprehensive multi−omics analysis of BALF, which integrates transcriptomic, metabolomic, microbiomics, and proteomic data, provides a powerful framework for systematically mapping the immune, metabolic, and microbial networks that drive the pathogenesis of CIP. The multidimensional information obtained through this approach can refine disease classification, facilitate early diagnosis, support individualized treatment decisions, and improve prognostic assessment. Looking ahead, expanding the number of clinical samples and carefully validating the relevance of potential biomarkers will be important steps toward translating these findings into practice. At the same time, future research should examine whether therapeutic strategies guided by multi-omics insights can lead to more effective prevention and management of CIP.

### 4.4 Challenges and limitations of BALF for CIP

#### 4.4.1 Standardization problems

The way BALF samples are collected, stored, and processed can strongly influence the results. Factors such as centrifugation speed and storage time can change cell counts and their classification. These changes can reduce diagnostic accuracy. In the diagnosis of CIP, standardized procedures are very important to reduce differences caused by individual operators. At present, there is no clear agreement on the normal range of BALF lymphocyte proportion, which has been reported to vary between 15% and 30%. Different institutions also use different reference criteria. Differences in lavage site and recovery volume can further affect the results (14). These variations make it difficult to compare data from different centers and show the urgent need for unified BALF handling.

#### 4.4.2 Limited specificity

BALF lymphocytosis is also observed in other interstitial lung diseases, such as sarcoidosis and hypersensitivity pneumonitis (14, 18), limiting its specificity for CIP diagnosis. Although BALF is frequently used to rule out infections, CIP itself may be associated with alterations in the respiratory microbiome, complicating interpretation. Pathogens such as influenza virus and *Pneumocystis jirovecii* have been detected in the BALF of CIP patients, yet it often remains unclear whether these findings indicate co-infection or reflect part of the CIP-related inflammatory process, potentially delaying targeted therapy (12). Microbiological analyses following antibiotic exposure may be further confounded by contamination, reducing diagnostic reliability (86, 87).

#### 4.4.3 Contraindications for BALF in the evaluation of CIP

The absolute contraindications of BALF collection include situations where the risk is unacceptably high, such as severe respiratory failure requiring substantial ventilatory support, significant airway obstruction, or active massive hemoptysis. In these cases, attempting BALF collection may precipitate rapid deterioration and should be avoided. This limitation reduces the use of BALF in certain patient groups (88). Other circumstances fall into the category of relative contraindications, where the potential risks and benefits must be weighed individually. Examples include moderate hypoxemia, unstable cardiovascular status, known bleeding disorders, or recent thoracic surgery. Here, the decision to proceed should ideally be discussed within a multidisciplinary team to ensure patient safety.

#### 4.4.4 Unclear value in long-term monitoring

At present, prospective evidence regarding BALF−derived biomarkers, such as T cell subsets and cytokine profiles, in evaluating treatment response in CIP remains scarce. In patients with pre−existing interstitial lung disease, the prognostic or predictive value of BALF for disease severity and therapeutic outcomes has not yet been clearly established. As a result, corticosteroid regimens are frequently formulated without the benefit of BALF based guidance. These limitations point to a notable gap in current clinical practice and emphasize the need for future studies to investigate the role of BALF in long−term monitoring, with particular attention to its potential in guiding treatment adjustments and predicting patterns of relapse or recovery.

## 5 Conclusions and perspective

### 5.1 Multicenter and standardized BALF analysis

Future research should prioritize multicenter collaboration and the standardization of BALF sample analysis procedures. Currently, substantial variability exists among centers in terms of sampling site, lavage volume, cell counting methods, centrifugation protocols, and storage conditions, which significantly impacts data comparability and reproducibility. To address these issues, unified guidelines for sample collection, processing, cryopreservation, and testing should be developed, supported by robust quality control systems and standardized reference workflows. Furthermore, recruiting BALF samples from diverse control cohorts, for example healthy volunteers and lung cancer patients treated with immunotherapy, could greatly enhance the representativeness of a reference database and strengthen the comparative value of future studies. Such a database should include baseline cytological and molecular parameters across different populations (e.g., age, sex, smoking status) to inform diagnostic thresholds and classification schemes for CIP.

### 5.2 Multi-omics BALF analysis and prediction models

Studying BALF at only one omics level cannot show the full complexity of CIP. Future research should combine clinical data with radiomics, transcriptomics, proteomics, metabolomics, and single−cell sequencing to build multi−omics models. Artificial−intelligence and machine−learning tools can help find links between BALF molecular patterns and clinical outcomes. These models may support early diagnosis and risk assessment in CIP. Systems that track molecular changes in BALF over time can also help doctors monitor disease progress and treatment response. With these advances, BALF could become not only a diagnostic tool but also a platform for precise prediction and treatment monitoring.

### 5.3 Mechanistic studies and personalized therapy

Future work should study immune cells and related molecular pathways in BALF to understand how CIP develops. Methods such as single−cell omics, spatial transcriptomics, and flow cytometry can show how the BALF immune environment changes during disease. These methods can also help find harmful cell types, like Tfh cells and CD8+ T cells, and describe their signaling. Researchers should also examine metabolic products and inflammatory lipids in BALF, as they may serve as treatment targets. Based on these findings, new therapies can be designed to adjust immune−cell activity, reduce inflammation, and support lung healing. Larger sample collections, better data−sharing, and studies across different regions and diseases are needed to improve reliability and make BALF findings useful in precision medicine.

## 6 Conclusion

Bronchoalveolar lavage fluid provides a valuable window into the immune processes underlying immune checkpoint inhibitor related pneumonitis. Current research shows its promise for improving diagnosis, monitoring, and treatment design. However, differences in sampling and analysis still limit consistency across studies. Looking ahead, stronger standardization and the integration of advanced analytical approaches may help transform BALF into a practical tool for precision diagnosis and personalized therapy in patients with CIP.
